# Enhancing Ethiopian power distribution with novel hybrid renewable energy systems for sustainable reliability and cost efficiency

**DOI:** 10.1038/s41598-024-61413-8

**Published:** 2024-05-10

**Authors:** Takele Ferede Agajie, Armand Fopah-Lele, Isaac Amoussou, Baseem Khan, Mohit Bajaj, Ievgen Zaitsev, Emmanuel Tanyi

**Affiliations:** 1 Department of Electrical and Electronic Engineering, Faculty of Engineering and Technology, University of Buea, PO.Box. 63, Buea, Cameroon; 2 Department of Electrical and Computer Engineering, Debre Markos University, Debre Markos, Ethiopia; 3 Department of Mechanical Engineering, Faculty of Engineering and Technology, University of Buea, PO.Box. 63, Buea, Cameroon; 4 Department of Electrical and Computer Engineering, Hawassa University, Hawassa, Ethiopia; 5 Department of Electrical Engineering, Graphic Era (Deemed to be University), Dehradun, 248002 India; 6 Hourani Center for Applied Scientific Research, Al-Ahliyya Amman University, Amman, Jordan; 7 Graphic Era Hill University, Dehradun, 248002 India; 8 Department of Theoretical Electrical Engineering and Diagnostics of Electrical Equipment, Institute of Electrodynamics, National Academy of Sciences of Ukraine, Peremogy, 56, Kyiv-57, 03680 Ukraine; 9 Center for Information-Analytical and Technical Support of Nuclear Power Facilities Monitoring of the National Academy of Sciences of Ukraine, Akademika Palladina Avenue, 34-A, Kyiv, Ukraine

**Keywords:** Power losses, Voltage deviation, Distribution system, GHG emissions, Grid-connected, Energy science and technology, Engineering, Mathematics and computing

## Abstract

Economic development relies on access to electrical energy, which is crucial for society’s growth. However, power shortages are challenging due to non-renewable energy depletion, unregulated use, and a lack of new energy sources. Ethiopia’s Debre Markos distribution network experiences over 800 h of power outages annually, causing financial losses and resource waste on diesel generators (DGs) for backup use. To tackle these concerns, the present study suggests a hybrid power generation system, which combines solar and biogas resources, and integrates Superconducting Magnetic Energy Storage (SMES) and Pumped Hydro Energy Storage (PHES) technologies into the system. The study also thoroughly analyzes the current and anticipated demand connected to the distribution network using a backward/forward sweep load flow analysis method. The results indicate that the total power loss has reached its absolute maximum, and the voltage profiles of the networks have dropped below the minimal numerical values recommended by the Institute of Electrical and Electronics Engineers (IEEE) standards (i.e., 0.95–1.025 p.u.). After reviewing the current distribution network’s operation, additional steps were taken to improve its effectiveness, using metaheuristic optimization techniques to account for various objective functions and constraints. In the results section, it is demonstrated that the whale optimization algorithm (WOA) outperforms other metaheuristic optimization techniques across three important objective functions: financial, reliability, and greenhouse gas (GHG) emissions. This comparison is based on the capability of the natural selection whale optimization algorithm (NSWOA) to achieve the best possible values for four significant metrics: Cost of Energy (COE), Net Present Cost (NPC), Loss of Power Supply Probability (LPSP), and GHG Emissions. The NSWOA achieved optimal values for these metrics, namely 0.0812 €/kWh, 3.0017 × 10^6^ €, 0.00875, and 7.3679 × 10^6^ kg reduced, respectively. This is attributable to their thorough economic, reliability, and environmental evaluation. Finally, the forward/backward sweep load flow analysis employed during the proposed system’s integration significantly reduced the impact of new energy resources on the distribution network. This was evident in the reduction of total power losses from 470.78 to 18.54 kW and voltage deviation from 6.95 to 0.35 p.u., as well as the voltage profile of the distribution system being swung between 1 and 1.0234 p.u., which now comply with the standards set by the IEEE. Besides, a comparison of the cost and GHG emission efficiency of the proposed hybrid system with existing (grid + DGs) and alternative (only DGs) scenarios was done. The findings showed that, among the scenarios examined, the proposed system is the most economical and produces the least amount of GHG emissions.

## Introduction

Reliable and sustainable access to electrical energy is crucial for socioeconomic progress and the welfare of people globally. Nevertheless, some areas, such as particular regions in Ethiopia, encounter substantial obstacles in fulfilling their energy requirements. Ethiopia’s Debre Markos distribution network has had regular power outages, with an average of more than 800 h per year in the last 5 years. These power failures interrupt daily activity and result in significant financial losses for businesses and homes. In order to address these difficulties, there is an increasing focus on shifting towards renewable energy sources that provide both environmental sustainability and improved reliability. Renewable energy technologies, such as solar photovoltaic (PV), biogas, and energy storage systems (ESS), offer practical solutions to increase the variety of energy sources and enhance energy security. Incorporating these technologies into the current electricity infrastructure can contribute to grid stability, decrease reliance on fossil fuels, and alleviate greenhouse gas emissions.

Renewable energy systems and energy storage technologies have made substantial progress in recent years, resulting in greater cost-effectiveness and practicality for broader implementation. Nevertheless, the efficient incorporation and supervision of these systems into current distribution networks necessitate meticulous strategizing, taking into account diverse technical, economic, and environmental aspects.

Ethiopia, a nation with significant economic potential and a growing population, has faced chronic power shortages that impact its development. The country’s electricity is predominantly generated through hydroelectric power, which, while renewable, presents challenges due to seasonal variability in rainfall and river flow. This dependency on hydroelectric power makes Ethiopia vulnerable to droughts, which have become more frequent and severe due to climate change. The government has embarked on ambitious projects like the Grand Ethiopian Renaissance Dam (GERD) to increase its power generation capacity. However, the realization of such projects has been slow and fraught with political and technical challenges, leaving the existing infrastructure strained and often unable to meet domestic demand.

Debre Markos, a city in the Amhara Region, epitomizes the struggles faced by many Ethiopian urban centers regarding electricity distribution. The city’s power infrastructure suffers from several critical issues. The distribution networks in Debre Markos are often outdated and not well maintained, leading to frequent and unpredictable power outages. The existing infrastructure does not have the capacity to handle peak loads, especially during times when demand spikes. This insufficiency is exacerbated by rapid urban growth and increased industrial activity. Inadequate infrastructure and outdated technology lead to high technical losses. These losses are due to energy dissipation in electrical system components like transformers and transmission lines, which is worsened by poor maintenance and overloading of the system. These are losses mainly due to theft (illegal connections to the grid) and inefficiencies in billing and collection processes. These practices not only cause revenue losses but also put additional strain on the power supply by increasing the apparent demand on the network.

The consequences of power outages in Debre Markos extend beyond mere inconvenience, leading to significant financial and resource losses. Frequent outages disrupt business operations, leading to loss of productivity and revenue. Small and medium enterprises, which often cannot afford backup power solutions like generators, are particularly hard-hit. Critical facilities like hospitals and schools suffer immensely, affecting service delivery and quality of life. For instance, power outages in hospitals can lead to life-threatening situations when life support machines and other critical medical devices fail. To cope with power unreliability, many businesses and institutions invest in diesel generators. This not only increases operational costs due to high fuel prices but also contributes to environmental pollution. The inefficiencies in the power distribution system lead to a significant waste of resources. Energy that could be used to power homes and businesses is lost in transmission, reflecting poor utility of capital and resources already scarce.

The power shortages and the resultant challenges in Debre Markos highlight a critical need for Ethiopia to upgrade its power infrastructure, diversify its energy sources, and improve maintenance and operational efficiencies. Addressing these issues is crucial for stabilizing the country’s power supply and supporting its economic growth aspirations. The situation in Debre Markos serves as a microcosm of the broader challenges facing Ethiopia’s power sector, necessitating urgent and comprehensive reforms to ensure energy security and sustainability.

### Motivations of this paper

Studying the impact of the integration of optimally sized hybrid renewable power generation on distribution networks is a valuable endeavor that can provide insights into various aspects of the energy system. Here are some potential motivations for such a study:

- *Renewable energy integration challenges* Explore the challenges associated with integrating renewable energy sources into distribution networks, especially when dealing with a hybrid system combining multiple sources like solar, wind, and possibly energy storage.
- *Grid resilience and reliability* Assess the impact on the overall resilience and reliability of distribution networks. Understand how the variability and intermittency of renewable sources affect the stability of the grid. Furthermore, it investigates the role of energy storage technologies in enhancing the reliability and stability of distribution networks with hybrid renewable systems. Assess the economic viability and efficiency of storage solutions.
- *Optimal sizing for efficiency* Investigate the optimal sizing of hybrid renewable power generation systems to maximize energy production while considering the capacity and constraints of distribution networks.
- *Techno-economic analysis and environmental benefits* Conduct a techno-economic analysis to evaluate the cost-effectiveness of integrating hybrid renewable systems. Assess the initial capital costs, operational and maintenance costs, and potential savings over time. Examine the environmental benefits associated with integrating renewable energy, including reducing greenhouse gas emissions and the overall environmental impact of the distribution network.
- *Grid management and control strategies* Explore strategies for effective grid management and control in the presence of hybrid renewable systems. This includes studying advanced control mechanisms, demand response, and grid-balancing techniques. In addition, it explores how integrating hybrid renewable systems aligns with broader grid modernization initiatives. Identify synergies and areas for collaboration with innovative grid technologies.
- *Impact on distribution equipment* Investigate the effects of renewable energy integration on distribution equipment such as transformers, switchgear, and protection devices. Understand potential stress and degradation factors.
- *Case studies and best practices* Analyze case studies of existing projects to identify best practices and lessons learned in integrating optimally sized hybrid renewables with energy storage power generation into distribution networks. This study can contribute valuable insights to the ongoing efforts to transition towards a more sustainable and resilient energy infrastructure by addressing these motivations.

### Related works

Incorporating optimally sized hybrid renewable power generation into distribution networks has been a topic of thorough investigation and analysis in renewable energy and power engineering fields. This field of research focuses on the difficulties and advantages of integrating various sustainable energy sources, such as solar and biogas, with SMES and PHES energy storage systems into conventional power grids.

Regarding the rising importance of sustainability and the need to decrease carbon emissions, there is a growing emphasis on incorporating renewable energy sources into the current electricity infrastructure^1–4^. Renewable sources, like solar and wind, are characterized by their intermittent and variable nature, which presents challenges for seamless integration into power grids^5,6^. Babatunde et al.^7^ described hybrid renewable power systems that combine multiple renewable energy sources to improve overall system reliability and efficiency. Li et al.^8^ and Liu et al.^9^ were presented by integrating complementary sources, such as solar and wind; a hybrid system can mitigate the intermittency and variability issues associated with individual sources. The variability and unpredictability of renewable energy sources can affect the stability and resilience of distribution networks. Researchers explore advanced control strategies, energy storage solutions, and smart grid technologies to enhance the grid’s ability to accommodate renewable energy fluctuations. Furthermore, implementing energy storage systems are another way, such as batteries, allows excess energy generated during peak times to be stored and used during periods of low generation^10–14^. This helps smooth out fluctuations and ensures a more consistent energy supply to the connected loads from the distribution networks.

The integration of renewable energy sources affects the operation and performance of distribution networks. Issues such as voltage regulation, power quality, and grid stability become important considerations when adding variable and distributed energy sources to the network. Technological advances, such as smart grids, energy storage systems, and advanced control algorithms, play a crucial role in facilitating the integration of renewable energy into distribution networks. These technologies facilitate improved monitoring, control, and management of distributed energy resources, thereby contributing to a more efficient and reliable energy system. The issue of cost-effectiveness is paramount in the integration of renewable energy sources. Consequently, researchers are actively engaged in evaluating the economic feasibility of hybrid systems and delving into various financing mechanisms aimed at incentivizing their widespread adoption and deployment.

Determining the optimal size of hybrid renewable power systems is crucial for maximizing their benefits^15–20^. Researchers focus on developing models and algorithms to optimize the sizing and design of hybrid systems based on factors like energy demand patterns, resource availability, and economic considerations.

Accurately determining the appropriate scale for hybrid renewable power generation systems is fundamental to realizing both the efficiency and cost-effectiveness of renewable energy resource utilization. Both software tools and metaheuristic optimization techniques play significant roles in this process^21–26^.

#### Software tools

- *System simulation software* Tools such as HOMER (Hybrid Optimization Model for Electric Renewables) and RET-Screen are extensively employed for simulating and optimizing hybrid renewable energy systems^27,28^. These tools consider various factors such as energy demand, renewable resource availability, and component specifications to determine the optimal system configuration.
- *Mathematical programming software* Optimization models are commonly structured using mathematical programming languages such as AMPL (A Mathematical Programming Language) or GAMS (General Algebraic Modeling System). These sophisticated tools possess the capability to resolve complex mathematical models, thereby facilitating the identification of the optimal component sizing within a hybrid renewable energy system^29,30^.
- *CAD software* Computer-aided design (CAD) tools are used to design the layout and integration of different renewable energy components in a hybrid system^31,32^. This includes the placement and configuration of solar panels, wind turbines, and energy storage systems^33–35^.

#### Metaheuristic optimization techniques

- *Genetic algorithms (GAs)* Genetic algorithms (GAs) derive inspiration from the principles of natural selection and genetics. By simulating this evolutionary process, GAs seek to iteratively refine potential solutions to ultimately arrive at the optimal sizing of components within a given system. GAs are particularly useful when dealing with an ample search space and non-linear optimization problems that happen during the sizing of hybrid renewable energy sources^36–38^.
- *Particle swarm optimization (PSO)* Particle swarm optimization (PSO) is inspired by the social behavior of birds or fish. In PSO, a population of particles, each representing a potential solution, moves through the solution space to find the optimal solution. PSO is suitable for both continuous and discrete optimization problems and is often employed in the optimal sizing of hybrid renewable energy systems^39,40^.
- *Simulated annealing (SA)* Simulated annealing (SA) emulates the annealing process in metallurgy, which involves heating a material and gradually cooling it to optimize its structure and remove defects. In optimization, SA navigates the solution space by permitting uphill moves with a diminishing probability, aiding in the escape from local optima during the sizing of each component in a hybrid renewable energy system^41,42^.
- *Whale optimization algorithm (WOA)* The Whale optimization algorithm (WOA) is a nature-inspired optimization algorithm designed to simulate the social behavior of humpback whales. It is used to find optimal solutions for optimization problems, and it can be applied to various fields, including the sizing of hybrid renewable power generation systems^43–45^.

Metaheuristic algorithms are remarkably versatile and can be applied to a wide array of problem domains due to their adaptability and flexibility. Hybrid renewable power generation system sizing involves complex, nonlinear, and often multi-objective optimization problems. Metaheuristics can handle a wide range of problem types without requiring specific problem formulations, making them well-suited for the diverse and dynamic nature of renewable energy systems^22,23,46^. The choice between them depends on the specific characteristics of the problem, the complexity of the system, and the available computational resources.

In general, the study of the impact of optimally sized hybrid renewable power generation on distribution networks encompasses a broad range of technical, economic, and environmental aspects. Researchers aim to address the challenges associated with integrating renewable energy sources while maximizing the benefits of a cleaner and more sustainable energy infrastructure.

The literature analysis provided a thorough analysis of energy management issues and possibilities at Debre Markos University, highlighting a dependence on diesel generators that increases operational expenses and environmental harm. Renewable energy sources such as solar photovoltaic (PV) and biogas, as well as energy storage systems like pumped hydroelectric storage (PHES) and superconducting magnetic energy storage (SMES), are potential options. However, determining the best setup and operation for these systems in the university’s distribution network is currently unclear. Existing research does not thoroughly investigate the multi-objective optimization needed to identify the most cost-effective and environmentally friendly option. These gaps in the literature inspire a problem formulation: How can a hybrid renewable energy system (HRES) be properly integrated into Debre Markos University’s distribution network to reduce operating costs, power interruptions, and environmental pollution? How can multi-objective optimization techniques be applied to determine the best cost-effective and environmentally friendly HRES configuration? These issues serve as the foundation for the suggested research approach and analysis, which attempt to solve the identified shortcomings in Debre Markos University’s present energy management research.

### Contribution and organization of the paper

This research contributes to the current knowledge in several ways. The study assesses the proposed hybrid renewable energy system (HRES) and how it may be included into the distribution network of Debre Markos University. The study utilizes backward/forward sweep load flow analysis and metaheuristic optimization techniques to investigate various objectives functions and constraints in order to identify the most economical and ecologically friendly design for Hybrid Renewable Energy Systems (HRES). The research emphasizes the financial and environmental advantages of deploying the proposed system by comparing it to existing configurations such as Grid plus DG and simply DG.

The research also offers insights into how successful the proposed solution is at decreasing power losses and voltage variations in the distribution network. This involves a thorough analysis of the voltage profile and network performance with and without the HRES system, emphasizing the enhancements made through optimization.

The study also contributes to knowledge by assessing the economic viability and environmental impact of the proposed HRES. This includes an analysis of the cost of energy (COE), the levelized cost of electricity (LCC), and greenhouse gas (GHG) emissions. Through this analysis, the study identifies the proposed HRES as a more cost-effective and environmentally sustainable solution compared to existing systems.

Overall, this research adds to the knowledge base by providing a comprehensive evaluation of the proposed HRES integration into the distribution network of Debre Markos University. It offers valuable insights into the financial, technical, and environmental aspects of the proposed system, highlighting its potential to improve energy management and sustainability at the university.

The remaining part of the paper is organized as follows: “Overview of selected study area” section provides an overview of the selected study subject; “Proposed hybrid energy sources layout descriptions and mathematical modeling” section presents a suggested description and structure of the hybrid systems as well as their mathematical modeling; A proposed problem formulation and optimization are presented in “Formulation of problems and optimization” section; meta-heuristic optimization approaches are presented in “Meta-heuristic optimization techniques” section; results and discussions are described in “Results and discussions” section; and a conclusion is presented in “Conclusion and future research directions” section.

## Overview of selected study area

This study investigates Debre Markos feeder 4, a component of the Debre Markos distribution network, situated at coordinates 10.33° north and 37.71° east. The analysis is based on load data obtained directly from the current distribution network.

### Connected load assessment on Debre Markos Feeder 4

**Figure 1:**
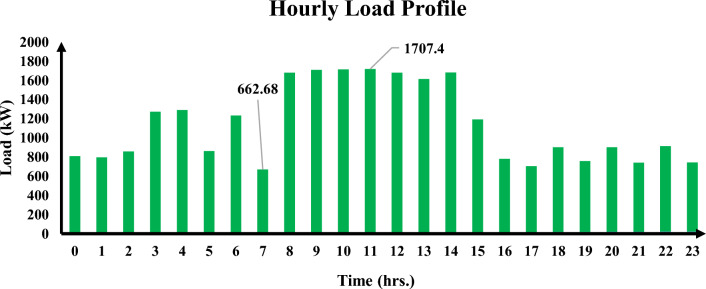
A community hourly load profile for the worst entire day.

Inadequate electricity is a problem in Debre Markos, a completely electrified town in northern Ethiopia. Residents are often forced to use diesel generators during prolonged power outages. A grid-connected hybrid renewable energy system was used in a case study to address this problem. The worst day load profile of the community grew throughout the day and decreased at night. The research indicates that the national grid is connected to the minimum load demands in the morning, underscoring the necessity of enhanced electrification in Ethiopia. As shown in Fig. 1, the maximum and minimum loads are connected to 1707.4 kWh and 662.68 kWh at 11:00 and 7:00 h, respectively.

### Resource assessment on the study area

The research case takes place in the northern Ethiopian city of Debre Markos. The best practices for sizing grid-connected hybrid solar PV and biogas systems with energy storage systems are assessed in this section. According to the data that is currently available, the average annual horizontal solar radiation is 6.67 kWh/m^2^/day, and the average ambient temperature is 18.5 °C. By combining primary and secondary data from multiple sources, we assessed the capacity of the site to generate biogas. The research region’s potential for producing biogas is assessed to be 7.93 × 10^6^ m^3^ per annum based on the data gathered. A rating power of 1293 kW and an annual energy generation of 11.33 × 10^6^ kWh might be achieved by using this biogas potential. Additionally, a pumped hydroelectric storage system for PHES consists of reservoirs connected by penstocks that are at various elevations. The upper reservoir’s source is the Chemoga River, which has average flow rates of 84.52 m^3^/s during the dry season and 463.94 m^3^/s during the rainy season. Stored energy can be calculated as the product of the elevation difference and the total mass of water.

## Proposed hybrid energy sources layout descriptions and mathematical modeling

**Figure 2:**
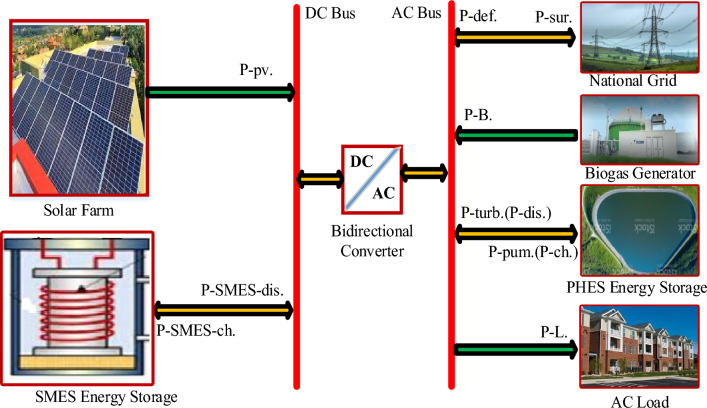
Schematic diagram of proposed grid connected hybrid system.

The schematic depiction of the proposed hybrid renewable energy system, which integrates solar photovoltaic (PV), biogas, and SMES-PHES energy resources, is illustrated in Fig. 2. This hybrid system is interconnected with the grid. Renewable energy sources primarily meet the demand for electricity, with the utility system serving as a backup only in the event of a power outage.

### Solar PV modeling

Ethiopia’s solar energy generation is largely based on insolation, with an average of 5.6011–6.80 kWh/m^2^, and can be integrated with temperature and sun irradiation. It would be simple to connect the temperature and solar irradiation with the power the solar-producing unit produces^47,48^.

1 $$ P_{PV} \left( t \right) = P_{PV - STC} \times f_{PV} \times \frac{{G_{T} \left( t \right)}}{1000} \times \left[ {1 + \alpha_{P} \left( {T_{C} - T_{C - STC} } \right)} \right], $$

2 $$ P_{PV - STC} = N_{PV} \times P_{\max . - STC}, $$

where P_PV_ stands for the PV panel’s power, 1000 for the solar irradiance at 25 °C during standard test conditions, G_T_ for the actual solar irradiance, T_C-STC_ is the operating cell temperature of the PV panel under standard test condition, T_C_ for the PV cell temperature, $\alpha_{p}$ is the power temperature coefficient, and f_PV_ for the PV’s derating factor.

### Biogas generator modeling

The biogas unit uses organic material fermentation to produce electrical energy, which is calculated using Eq. (3) and incorporated in power balancing computations using an easy-to-understand output power formula^49,50^:

3 $$ P_{B} \left( t \right) = \frac{{V_{B} \left( t \right) \times CV_{B} \times \eta_{B} }}{{860 \times t_{B} }}, $$

The variables utilized as decision-making variables in this system are $P_{B}$, $V_{B}$, $CV_{B}$, $\eta_{B}$ and $t_{B}$, and are represented the rated output power of the biogas generator (kW), the volume of biogas produced (m^3^/day), the calorific value of the available biogas (kcal/m^3^), the efficiency of the biogas system (%), and the number of working hours of the biogas system per day (h/day), respectively.

### PHES storage system unit modeling

This energy storage mechanism operates conventionally, constantly monitoring energy flow on the electrical network. When excess energy is present, it goes into pumping mode. When photovoltaic sources cannot meet demand, the system switches to power-generation mode. In order to carry out the pumping and generating modes of operation, the system’s energy balance must be correctly calculated using the following equation:

4 $$ P_{bala} \left( t \right) = P_{PV} \left( t \right) - P_{L} \left( t \right), $$

where $P_{bala}$, $P_{PV}$ and $P_{L}$ represents the power balance of the system, Power generated from solar, and connected load on the system, respectively.

#### Generating mode ($P_{bala} < 0$)

The PV system cannot provide enough electricity to meet the demand, necessitating energy from the storage system. The upper reservoir’s water capacity and turbine output power determine the power output, with the storage system attempting to function^51–54^:

5 $$ E_{PHES}^{gen} \left( t \right) = \min \left[ {\min \left( {\frac{{V_{{\left( {t - 1} \right)}} }}{3600};Q_{T} } \right)\eta_{T} \eta_{P} \rho g\left( {h_{add} + h_{3} } \right);\left| {E_{B} } \right|} \right], $$

6 $$ Q_{dis} \left( t \right) = \frac{{E_{PHES}^{gen} \left( t \right)}}{{g \times \rho \times \eta_{T} \times \eta_{P} \times \left( {h_{add} + h_{3} } \right)}}, $$

7 $$ P_{PHES}^{gen} \left( t \right) = Q_{dis.} \left( t \right) \times g \times \rho \times \eta_{T} \times \eta_{P} \times \left( {h_{add} + h_{3} } \right), $$

where $V_{{\left( {t - 1} \right)}}$,$\rho$,$\eta_{T}$,$\eta_{P}$, g, $Q_{dis}$, $\left( {h_{add} + h_{3} } \right)$, $E_{\left( t \right)}^{gen}$ and $\left| {E_{B} } \right|$ are stands for the volume of water available in the reservoir at t − 1, water density, the efficiency of the generation system, the efficiency of the pipeline, the gravitational constant (9.81 m/s), the flow rate of water discharge, the water level in the upper reservoir, the energy generated by PHES, and the energy balance of the system, respectively.

#### Pumping mode $\left( {P_{bala} > 0} \right)$

The suggested method pumps water using excess energy until the top reservoir is full, based on the reservoir’s water level, excess energy, and PHES pumping mode maximum power.

8 $$ E_{PHES}^{pump} \left( t \right) = \min \left[ {\min \left( {\frac{{V_{\max } - V_{{\left( {t - 1} \right)}} }}{3600};Q_{P} } \right)\frac{{\rho \times g \times \left( {h_{add} + h_{3} } \right)}}{{\eta_{pump} \times \eta_{P} }};\left| {E_{B} } \right|} \right], $$

9 $$ Q_{cha} \left( t \right) = E_{PHES}^{pump} \left( t \right) \times \left\{ {\frac{{\eta_{T} \times \eta_{P} }}{{g \times \rho \times \left( {h_{add} + h_{3} } \right)}}} \right\}, $$

10 $$ P_{PHES}^{pump} \left( t \right) = Q_{dis.} \left( t \right) \times \left\{ {\frac{{g \times \rho \times \left( {h_{add} + h_{3} } \right)}}{{\eta_{pump} \times \eta_{P} }}} \right\}, $$

where $V_{\max }$, $\eta_{Pump}$, $Q_{cha}$, $E_{PHES}^{pump}$ and $P_{PHES}^{pump}$ stands for the reservoir’s maximum capacity, the pumping system’s efficiency, the flow rate of charge water to the upper reservoir, and the amount of energy and power used during pumping, respectively.

### SMES storage system unit modeling

This energy storage mechanism stores excess energy from hybrid systems, releasing power when the generation can’t meet the connected load and allowing long-term energy sources to be connected in a rapid-response manner^55,56^. The two ways of operation of this energy storage technology are described below.

#### Charging mode ($P_{L} - P_{sys} < 0$)

This state of operation occurs when the hybrid system generation exceeds the load requirement.

11 $$ P_{exch} \left( t \right) = \max \left\{ { - \left| {\Delta P\left( t \right)} \right|,\frac{{\left( {(E_{exch} \left( {t - 1} \right) - E_{exch - \max } \left( t \right)} \right)}}{{\Delta t \times \eta_{ch} }}, - P_{exch - rated} } \right\}, $$

12 $$ E_{exch} \left( t \right) = \min \left\{ {\left( {E_{exch} \left( {t - 1} \right) - P_{exch - ch} \left( t \right) \times \Delta t \times \eta_{ch} } \right),E_{exch - \max } } \right\}. $$

#### Discharging mode ($P_{L} - P_{sys} > 0$)

When the load demand exceeds the hybrid system’s power, this mode of operation is activated.

13 $$ P_{exch} \left( t \right) = \max \left\{ {\left| {\Delta P\left( t \right)} \right|,\frac{{\left( {(E_{exch} \left( {t - 1} \right) - E_{exch - \min } \left( t \right)} \right) \times \eta_{dis} }}{\Delta t},P_{exch - rated} } \right\}, $$

14 $$ E_{exch} \left( t \right) = \max \left\{ {\left( {E_{exch} \left( {t - 1} \right) - \frac{{P_{exch,dis} \times \Delta t}}{{\eta_{dis} }}} \right),E_{exch - \min } } \right\}, $$

where $P_{exch}$, $\eta_{cha}$ and $\eta_{dis}$ represents the SMES exchanged power at period t, the charging and discharging efficiencies, respectively. In addition, $\Delta P\left( t \right)$, $P_{exch - rated}$, $E_{exch}$, $E_{exch - \min }$, $E_{exch - \max }$ and $\Delta t$ represents the difference between hybrid power system output and load demand, the SMES power rating, the SMES energy storage capacity at period t, minimum and maximum energy storage limits, and the time interval (1 h). The state of charge (SOC) is an indicator of the energy that is stored in the SMES. Each SMES that serves a distinct load model will have an initial SOC value that needs to be optimized^57–59^.

### Inverter energy conversion modeling

Equation (15) explains a power inverter that converts DC solar PV output into AC electricity, used in energy management strategies, with the inverter’s size calculated accordingly^60,61^:

15 $$ P_{inv} \left( t \right) \ge P_{PV} \left( t \right) \times \eta_{inv} \times N_{PV} \times f_{PV} + P_{SMES} \left( t \right), $$

where $P_{inv}$ and $\eta_{inv}$ represents the inverter output AC power and the power inverter efficiency (%), respectively.

## Formulation of problems and optimization

Proposing optimal sizing formulae for each parameter allows for the optimization of the cost, reliability, and CO_2_ emission function. The proposed grid-connected hybrid system’s best component sizes and how well it works depend on how well optimization problems are solved. In addition, the distribution power losses and voltage deviations are taken into consideration. These problems must be solved in order to find the proper generation unit sizes and numbers.

### Objective functions for optimal sizing of HRES units perspectives

Three objective functions are taken into consideration for this particular scenario, as described in the issue formulation section above: finance (NPC), reliability (LPSP), and GHG emissions (CO_2_ emissions). Equations (16)–(18) represent the objective function minimization in the aforementioned cases as follows:

16 $${F}_{1}={\text{min}}\left\{NPV\right\}={\text{min}}\left\{\frac{{C}_{ann-total}}{CRF}\right\}= {\text{min}}\left\{\frac{{C}_{ann-cap}+{C}_{ann-rep}+{C}_{ann-O\&M}+{C}_{GP}-{C}_{GS}}{\frac{r{\left(1+r\right)}^{n}}{{\left(1+r\right)}^{n}-1}}\right\},$$

17 $${F}_{2}={\text{min}}\left\{LPSP\right\}={\text{min}}\left\{\frac{{\sum }_{t=1}^{8760}\left[\begin{array}{c}\left({P}_{load}\left(t\right)+{P}_{PHES\&SMES-cha}\left(t\right)\right)-\\ \left({P}_{PV\&B}\left(t\right)+{P}_{PHES\&SMES-dis}\left(t\right)+{P}_{GP}\left(t\right)\right)\end{array}\right]}{{\sum }_{t=1}^{8760}{P}_{load}\left(t\right)}\right\},$$

18 $${F}_{3}={\text{min}}\left\{C{O}_{2}Emissions\right\}=\mathit{ }\text{ min}\left\{\begin{array}{c}{E}_{PV}\left(t\right)\times EE{F}_{PV}+{E}_{B}\left(t\right)\times EE{F}_{B}\\ +{E}_{PHES}\left(t\right)\times EE{F}_{PHES}+{E}_{SMES}\left(t\right)\times EE{F}_{SMES}\end{array}\right\}.$$

In addressing this issue, the optimization considerations involve a range of factors such as the number of solar panels, the capacity of the biogas generator, the maximum installation capacity of the inverter, the maximum installation capacity of SMES, the maximum installed power of PHES, and the sizes of the upper reservoirs. These constraints are applicable to the objective functions mentioned earlier. The optimization problem is further defined by the constraints outlined in Eq. (19).

19 $$ \left. {\begin{array}{*{20}l} {{\text{N}}_{{{\text{PV}}}}^{\min } \le N_{PV} \le {\text{N}}_{{{\text{PV}}}}^{\max } } \hfill \\ {P_{inv} \ge P_{PV}^{\max } } \hfill \\ {P_{B}^{\min } \le P_{B} \le P_{B}^{\max } } \hfill \\ {P_{SMES}^{\min } \le P_{SMES} \le P_{SMES}^{\max } } \hfill \\ {0 \le P_{GP} \le P_{GP}^{\max } } \hfill \\ {P_{SMES}^{\min } \le P_{PHES} \le P_{PHES}^{\max } } \hfill \\ {V_{{{\text{Reservoir}}}}^{\min } \le V_{{{\text{Reservoir}}}} \le V_{{{\text{Reservoir}}}}^{\min } } \hfill \\ \end{array} } \right\}, $$

where ${N}_{PV}^{min}$, ${N}_{PV}^{max}$, ${P}_{B}^{min}$, ${P}_{B}^{max}$ ${P}_{PHS}^{min}$, ${P}_{PHS}^{max}$, ${P}_{SMES}^{min}$, ${P}_{SMES}^{max}$, ${V}_{Reservoir}^{min}$, ${V}_{Reservoir}^{max}$ represent the maximum and minimum limits of the number of solar panels, the output power of biogas, PHES, and SMES, and the upper reservoir, respectively.

### Objective functions for the HRES integration impacts on distribution network perspective

The distribution network’s voltage variation and distribution power losses are reduced when optimal HRES integration is implemented into the system. If the right and optimal size of HRES fits specific requirements, it can be a viable solution to the issue.

The main impact of integrating HRES into the distribution system is to reduce real power loss and voltage deviation while taking various constraints into account. Thus, it has the following mathematical expression:

20 $${F}_{1}=Min.\left({P}_{Losses}\right)=Min.\left({\sum }_{i=1}^{m}({I}_{i}^{2}\times {R}_{i})\right),$$

21 $${F}_{2}=Min.\left({V}_{Deviation}\right)=Min.\left({\sum }_{i=1}^{m}{\left(1-{V}_{i}\right)}^{2}\right),$$

where P_Losse_, V_Deviation_, V_i_, I_i_, R_i_, i, and m are represented as power losses, voltage deviation, voltage magnitude at bus (node) i, current magnitude at bus (node) i, resistance magnitude at bus (node) i buses (nodes) and number of network buses (nodes) respectively.

The impact of HRES integration into the distribution system is to minimize the real power loss, and the voltage deviation is subjected to satisfy all of the operational constraints, such as limits of node voltages (${V}_{i}^{min}\le {V}_{i}\le {V}_{i}^{{\text{max}}}$), limits of HRES capacity (${S}_{\text{HRES}}^{min}\le {S}_{HRES}\le {V}_{HRES}^{{\text{max}}}$), limits of Branch thermal (${I}_{\left(i,j\right)}\le {I}_{rated}$), and limits of active power losses (${P}_{Losses}\text{with HRES}\le {P}_{Losses}\text{without HRES}$) before and after HRESs.

## Meta-heuristic optimization techniques

Two metaheuristic optimization algorithms were presented in this work to analyze the configuration of grid-connected hybrid renewable energy systems, as depicted in Fig. 5. These strategies are multi-objective particle swarm optimization (MOPSO) and the Non-dominated sorting whale optimization algorithm (NSWOA). Below is a description of these algorithms.

### Non-dominated sorting whale optimization algorithm (NSWOA)

The WOA is a distinctive metaheuristic optimization method that Mirjalili and Lewis developed with inspiration from nature^62^. WOA is typically comprised of a bubble-net attacking model, seeking prey, and enclosing prey. The coefficients that comprise NSWOA are as follows: (rs) is an arbitrary vector in (0, 1), (As) is a coefficient inside (− 1, 1), and (as) is a coefficient that linearly minimizes from 2-0 in each step^63,64^. The pseudo-code of NSWOA is presented in Algorithm 1.

**Algorithm 1:**
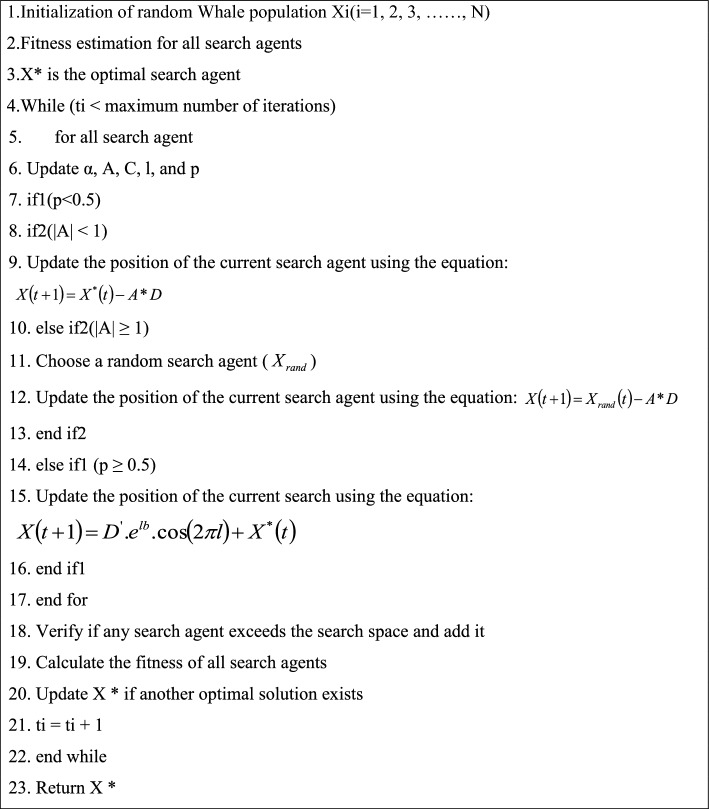
NSWOA.

### Multi-objective particle swarm optimization (MOPSO)

By utilizing intelligence and swarm placements, PSO is able to find the best solution^65^. When the number of iterations exceeds the maximum or the desired output is obtained, the searching process automatically ends^66^. When the MOPSO algorithm was originally put into practice, a population was started at random and updated using a velocity vector. The latter combined the best personal and global perspectives. On the other hand, the two places represent the best global resolution that each particle has to provide in terms of fitness. The MOPSO parameters include population size (n), personal learning coefficient (c1), global learning coefficient (c2), inertia weight (w), and inertia weight damping ratio (wdamp)^67,68^. Algorithm 2 shows the pseudo-code of MOPSO.

**Algorithm 2:**
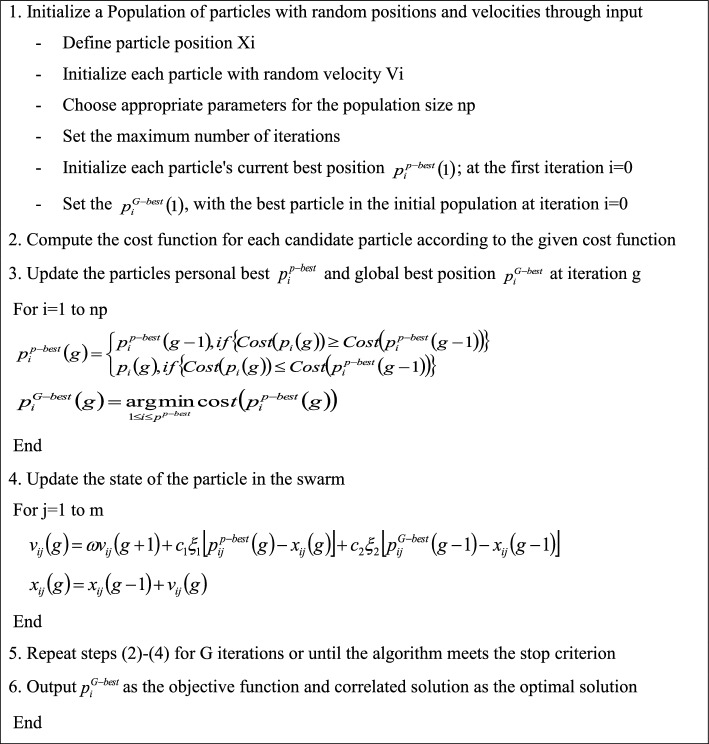
MOPSO.

## Results and discussions

### Optimal sizing of hybrid components

In this scenario, the decision variables are determined by the components of the grid-connected system, including the number of solar PV panels, the capacity of the biogas generator, the capacity of the PHES system, the capacity of the SMES system, and the capacity of the upper reservoir. We analyze three different optimization cases (NPC, LPSP, and CE) within a grid-connected HRES that utilizes a PHES-SMES energy storage system. The objective functions NPC, LPSP, and CE are each optimized to determine the optimal sizes of the HRES system’s components. Table 1 displays the capacities of the components that were optimized using a variety of metaheuristic optimization methods.

**Table 1:** Capacity of components considering three objective functions using two metaheuristic optimization techniques.

Techniques	Type of renewable energy resources				
	No of PV panel	PHES capacity (kW)	Reservoir capacity (m^3^)	Capacity of biogas (kW)	SMES capacity (kWh)
NSWOA	5986.35	450.67	35,298.14	860.19	142.28
MOPSO	5898.42	388.39	30,420.25	940.36	142.28

The PV modules, biogas generator, SMES, PHES, and upper reservoir have estimated capacities of 35,298.14 m^3^, 860.19 kW, 142.28 kWh, and 450.67 kW, respectively. Table 1 demonstrates that NSWOA is used to optimize the size. The MOPSO method was also utilized to determine the quantity of solar PV panels (5,899), the biogas generator’s capacity (940.36 kW), the PHES’s capacity (388.39 kW), and the upper reservoir’s capacity (30,420.25 m^3^). In contrast, SMES’s storage capacity remains constant throughout all optimization strategies at 142.28 kWh.

### Evaluation of cost-effective, reliability, and carbon emission factors

The Pareto front approach yields the best solution outcomes among multi-objective optimization techniques. The best answer to one objective in a multi-objective issue could be the worst answer to another. For example, the LPSP value may drop as the NPC numerical values rise. The initial cost of establishing the renewable energy system is increased due to the facility’s increased capacity, which also boosts the levelized cost of energy. The capacity of the components optimized employing two well-liked and chosen metaheuristic techniques is recorded in Table 2.

**Table 2:** Metaheuristic optimization techniques were used to optimize the evaluation parameters.

Evaluation parameters		Techniques	
		NSWOA	MOPSO
Financial	NPC (€)	3.0017 × 10^6^	3.010 × 10^6^
	LCOE (€/kWh)	0.052218	0.052985
	COE (€/kWh)	0.081201	0.082542
	LCC (€)	4.5071 × 10^6^	4.5151 × 10^6^
Reliability	EENS	1.1241 × 10^5^	1.1861 × 10^5^
	LPSP	0.008753	0.009648
	IR	0.999058	0.999008
	LOLP	2.327438	3.327275
	LOLE	9.706182	12.203275
GHG	Existing kgCO_2_	1.6122 × 10^7^	1.6122 × 10^7^
	HRES (kg${\text{CO}}_{2}$)	8.7536 × 10^6^	8.7945 × 10^6^
	KgCO_2_ reduction	7.3679 × 10^6^	7.3275 × 10^6^

**Figure 3:**
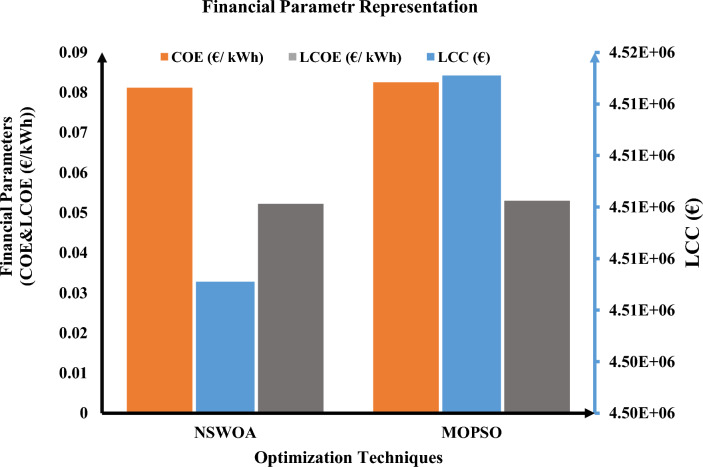
Financial parameters representation using proposed techniques.

Table 2 illustrates that the predefined LPSP value remains below 1% throughout all optimization methods. The results of the MOPSO algorithm and the NSWOA algorithm are nearly identical, with just minor numerical value changes. The entire project cost over a 25-year period is considered in this study. The total NPC, CO_2_ emissions, and LPSP are 3.0017 × 10^6^ €, 1.6122 × 10^7^ kgCO_2_, and 0.008753, respectively, in accordance with the outcomes of the NSWOA algorithm. The total NPC, CO_2_ emissions, and LPSP obtained by the algorithm are 3.010 × 10^6^ €, 1.6122 × 10^7^ kgCO_2_, and 0.009648 for MOPSO, respectively. Recorded results show that the current carbon emissions on the distribution network are all the same (1.6122 × 10^7^ kgCO_2_). However, the HRES CO_2_ emissions (kgCO_2_) obtained by using MOPSO and NSWOA are 8.7945 × 10^6^ and 8.7536 × 10^6^ kgCO_2_, respectively. When compared to the other two surviving algorithms, NSWOA has the best total NPC, LPSP, and GHG, according to the examination of these data. The NSWOA result is best for all values of financial parameters, including LCC, COE, and LCOE, as illustrated in Fig. 3.

**Figure 4:**
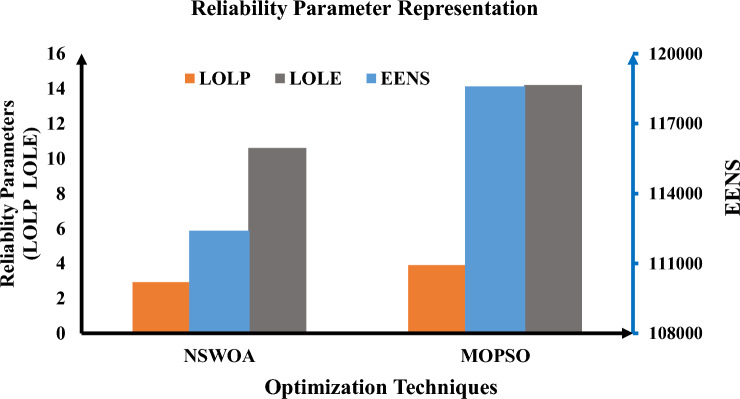
Reliability parameters representation using proposed techniques.

Moreover, the numerical values of the constraints, as well as the participation rate of renewable energy generation with energy storage systems, have a significant impact on the reliability analysis. As illustrated in Fig. 4, NSWOA is optimal for all values of reliability parameters such as LOLP, EENS, and LOLE.

**Figure 5:**
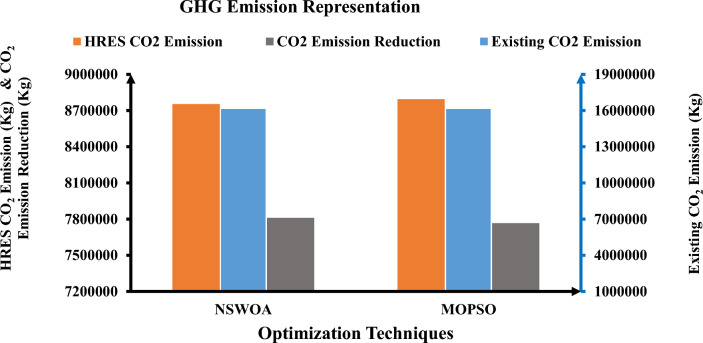
GHG emissions parameters representation using proposed techniques.

Furthermore, the numerical values of the limitations and the rate at which energy storage devices are coupled with renewable energy generation have a substantial influence on the GHG emissions analysis. NSWOA is optimal for all values of GHG emission parameters, including current CO_2_ emissions (kgCO_2_), PHRES (kgCO_2_), and PEA (kgCO_2_), as shown in Fig. 5.

### Analysis of HRES optimal solutions annually

**Figure 6:**
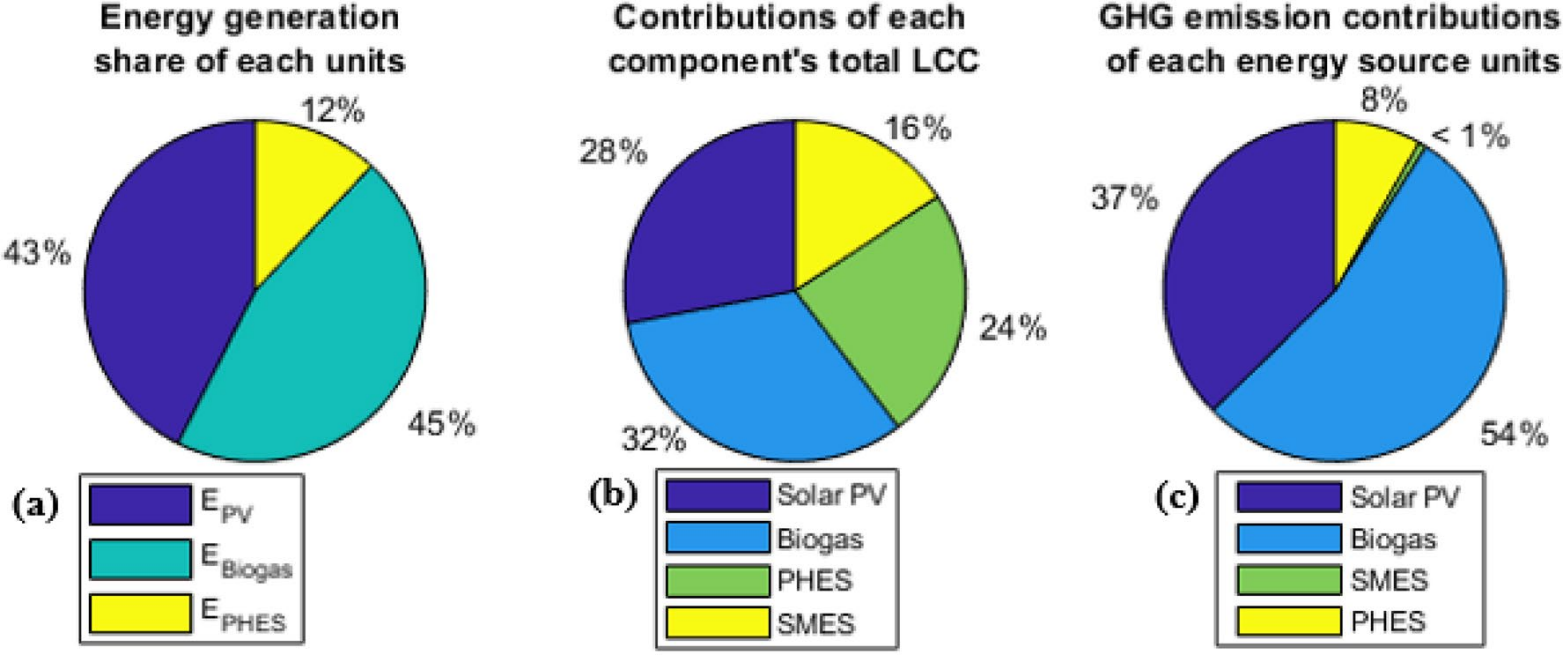
Contributions of each energy source (**a**), total life cycle cost (**b**), and greenhouse gas emissions (**c**) to the proposed hybrid system.

In this study, the NPC, LPSP, and CO_2_ emissions are the criteria, and the objective functions are employed to make decisions. The remaining studies focused on the NSWOA results based on the outcomes of the MOPSO metahirustic techniques. Figure 6a presents the energy distribution for the connected system load and the respective contributions from the hybrid renewable energy system’s (HRES) components to the energy storage system. Within the hybrid solar PV–biogas with SMES–PHES energy storage project, the PV system contributes 4.1258 × 10^6^ kWh, representing 43% of the total installed energy, while the biogas generator system accounts for 4.4154 × 10^6^ kWh, or 45% of the total capacity. Additionally, PHES generates 1.1582 × 10^6^ kWh, which equates to 12% of the hybrid system’s total installed capacity. It is essential to note that SMES, generating 1.315 × 10^5^ kWh, is primarily dedicated to maintaining steady peak transition demands during power transitions and is not considered a long-term energy source.

Figure 6b displays the entire life cycle cost of the project as well as the relative contributions of each HRES component to the energy storage system. In the hybrid solar PV-biogas with SMES-PHES energy storage project, the PV system accounts for 1.2838 × 10^6^ € (28%) of the total project costs, while the biogas generating system accounts for 1.4757 × 10^6^ € (32%). Furthermore, the SMES and PHES account for 7.1853 × 10^5^ € (16%) and 1.0941 × 10^6^ € (24%), respectively, of the project’s total expenses. Figure 6c displays the system’s greenhouse gas emissions as well as the contributions made by each HRES component to the energy storage system. The hybrid solar PV-biogas with SMES-PHES energy storage project results in 3.1459 × 10^6^ kg CO_2_ emissions from the PV system (37.33%) and 4.5258 × 10^6^ kg CO_2_ emissions (54%) from the biogas generator system. Moreover, the SMES and PHES had CO_2_ emissions of 0.6949 × 10^6^ kg (8%) and 0.06082 × 10^6^ kg (0.73%), respectively. The power differential between the connected load and renewable power generation, biogas generators, and solar PV-generated energy conversions was 2.9585 GWh, 4.4154 GWh, and 4.8958 GWh annually, respectively. The power differential between the connected load and renewable power generation, as well as the highest and minimum values of the solar PV and biogas generator, were 1.6874 and 0 MW, 0.8603 and 0 MW, and 2.5484 and 0 MW, respectively. The result in Table 3 shows how much energy the power plant’s energy source units produce and use annually.

**Table 3:** Annual capacities and energy consumed and generated by the energy source units of the power plant by utilizing NSWOA techniques.

Hybrid system components and parameters				Numerical values
Connected loads		Energy consumed by the loads (GWh/year)		9.2664 GWh
		Maximum rated power of the loads (MW)		1.7074 MW
		Minimum rated power of the loads (MW)		0.66268 MW
Solar PV		Generated energy from solar PV (GWh/year)		4.8958 GWh/year
		Maximum rated power of the solar PV (MW)		2.5484 MW
Biogas generator		Generated energy by biogas generator (GWh/year)		4.4154 GWh/year
		Maximum rated power of the generator (MW)		0.8603 MW
PHES	Generated mode	Energy generated by the turbine (GWh/year)		1.14567 GWh/year
		Maximum rated power of the turbine (MW)		0.45067 MW
	Pumped mode	Energy consumed by the pump (GWh/year)		0.9667 GWh/year
		Maximum rated power of the pump (MW)		0.3803 MW
SMES		Discharge energy From SMES (GWh/year)		0.1236 GWh/year
		Consumed energy by SMES (GWh/year)		0.1544 GWh/year
		Maximum rated power of SMES (MW)		0.1186 MW
		SOC of SMES (%)	Minimum	5%
			Maximum	100%
Upper reservoir		Discharged water (m^3^/year)		7.325 × 10^6^ m^3^/year
		Charged water (m^3^/year)		7.543 × 10^6^ m^3^/year
		SOC of upper reservoir (%)	Minimum	61%
			Maximum	100%
Surplus		Surplus energy (GWh/year)		0.1352 GWh/year
		Maximum rated power of surplus (MW)		0.886 MW
Deficit		Deficit energy (GWh/year)		0.0281 GWh/year
		Maximum rated power of deficit (MW)		0.006987 MW

In Table 3, annual energy surplus and deficit in relation to energy exchange between the national utility grid and the hybrid system are reported for specific LPSP values. A surplus of energy is recorded when solar PV generation surpasses load demand and storage systems (SMES and PHES) are at full capacity. Conversely, a deficit occurs when load demand exceeds generation from solar PV and biogas systems and the water level in the PHES upper reservoir reaches its minimum level. The surplus and deficit energy of the hybrid system amount to approximately 0.1352 GWh and 0.0281 GWh, respectively, and its peak power is measured at 0.886 and 0.006987 MW. Depending on the energy surplus or deficit, the power exchange with the national utility grid was equal to the energy surplus sent to the national utility distribution network. The maximum connected loads in this table determine how the SMES’s discharging power, charging power, and state of charge will fluctuate over time. Only when each energy resource changes to meet the load does this energy storage system operate. Its response time is less than five milliseconds, so frequency and power deviation won’t be an issue. The SMES energy storage systems have an annual energy discharge and charge of 0.1236 GWh and 0.1544 GWh, respectively. The batteries range in level of charge from 5 to 100%. Moreover, Table 3 shows how much energy is used in the solar PV generation process and how much extra energy is available for use in the hybrid system that is being suggested. This graph visually represents the annual activities of the PHES energy storage system, including the quantity of water pumped into the upper reservoir during the pumping mode, the water used or discharged during power generation, the energy consumed during the changing mode, and the energy generated during discharging. It also provides information on the SOC parameter, which controls the water level in the upper reservoir. The PHES energy storage system’s activities involve generating, discharging, charging, and consuming energy at rates of 1.14567 GWh and 0.9667 GWh annually, respectively. The SOC of the upper reservoir represents the difference in water volume between its full and empty states. During periods of low PV energy, the upper reservoir’s SOC can decrease to a minimum of 61%. Annually, the upper reservoir is charged and discharged with 7.543 × 10^6^ and 7.325 × 10^6^ m^3^ of water, respectively.

### Dissections on impacts of HRES integration on distribution network

Distribution power losses and voltage deviation for the distribution network are reduced by the inclusion of an optimum-sized HRES in the local utility distribution system. If the right and ideal size of HRES fits specific requirements, it can be a viable solution to the issue. The primary effects of integrating HRES into the distribution system, subject to various limits, are to reduce power loss and voltage deviation.

**Figure 7:**
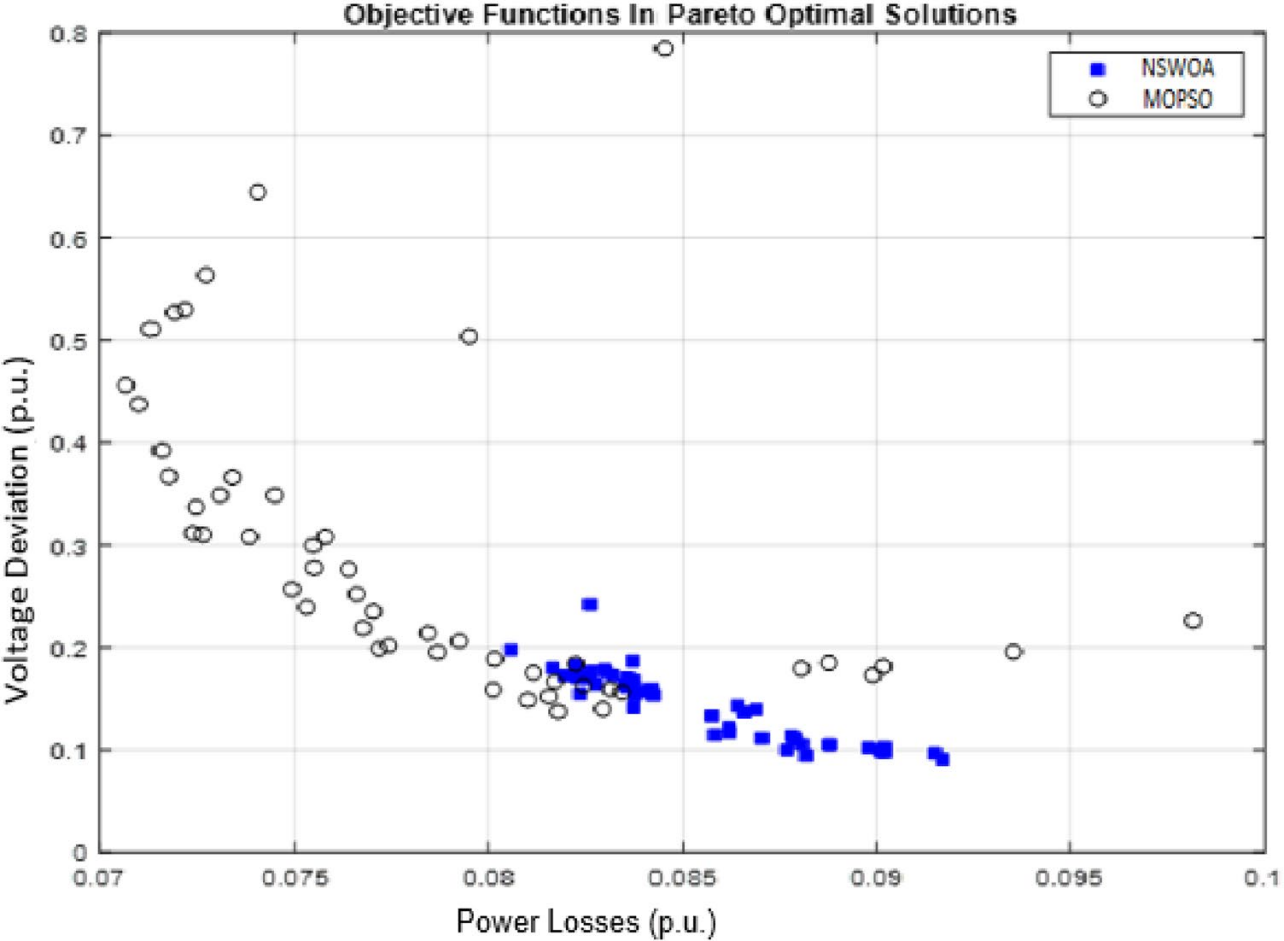
Performance comparison of NSWOA with MOPSO algorithms.

**Figure 8:**
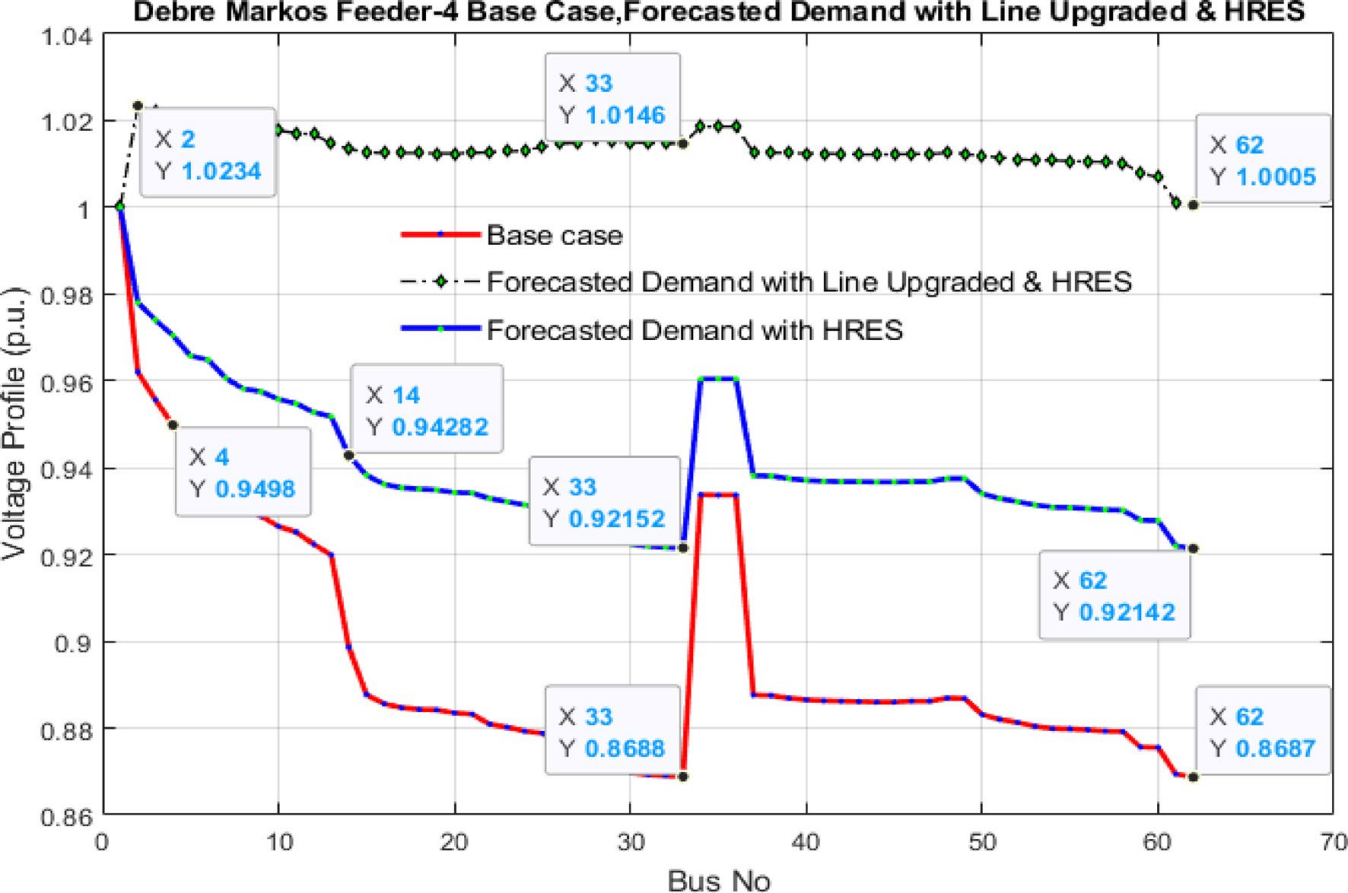
Comparison of the voltage profile of the system in the base case, with HRES system and line upgraded with HRES system by using NSWOA technique.

In this distribution network system, the proposed algorithm (NSWOA) is compared to MOPSO in terms of performance. This comparison is displayed in Fig. 7, and it is accomplished by estimating the spread and convergence time of the algorithm. The MOPSO algorithm was selected as the comparison method because it is simple to construct and can achieve rapid convergence, in contrast to similar multi-objective meta-optimistic optimization techniques. With the help of the proposed NSWOA over MOPSO, a successful search for the best optimal solution with regard to all objective functions was carried out, as indicated by the findings in Fig. 7. Hence, minimum numerical values of total power losses and voltage deviations are obtained by using NSWOA. Total power losses are denoted by the symbol Ploss, while total voltage variation is denoted by the symbol VD. Table 4 displays the load flow outcome both with and without the HRES situation. HRES is suggested as a way to improve the distribution line capacity as well as lessen the system’s issues with voltage variation and power loss. The load flow result shown in Table 4 is achieved once HRES has been introduced into the system. Figure 8 displays the node voltage profile of the base-case system under study.

**Table 4:** Power losses and voltage deviation with and without HRES system.

Cases	Total ploss (kW)	Total VD (p.u.)
Feeder-4 base case	476.61	7.983
Feeder-4 with HRES	85.54	1.45
Feeder-4 with line upgraded and HRES	18.54	0.35

As illustrated in Fig. 8, in the base case, the voltage profile for nodes 4 to 62 falls below 0.95 p.u., which is lower than the IEEE’s standard voltage profile values^69,70^. The voltage at node 62 is 0.8687 p.u. Meanwhile, in the case of forecasted electricity demand supplied by the HRES system, it also faced the same problem on the voltage profile as the base case. At this voltage level, electronic appliances connected to this node cannot function properly, which affects the lifespan of the equipment. This suggests that the system requires enhancements to improve its voltage profile and alleviate power losses linked to voltage drops within the system. Nonetheless, when the forecasted electricity demand is fulfilled by the HRES system, and the local grid distribution line capacity is upgraded, this leads to the reduction of power losses and an enhancement in the voltage profile of the distribution network. As shown in Fig. 8, in this case, nodes 1 to 62 have a voltage profile swing between 0.95 and 1.025 p.u. that fulfills IEEE standards.

The optimal sizing of HRES is executed using NSWOA and MOPSO, and the results are recorded in “Optimal sizing of hybrid components” section in Table 1. The reduction of voltage deviation and power losses by optimally sizing HRESs with the use of MOPSO and NSWOA is presented in Table 5.

**Table 5:** Results of total power loss and voltage deviation using NSWOA and MOPSO.

Feeder	Optimization techniques	Total power loss (kW)	Total voltage deviation (p.u.)
Debre Markos Feeder-4	Base case	470.78	6.95
	NSWOA	18.54	0.35
	MOPSO	24.75	0.56

**Figure 9:**
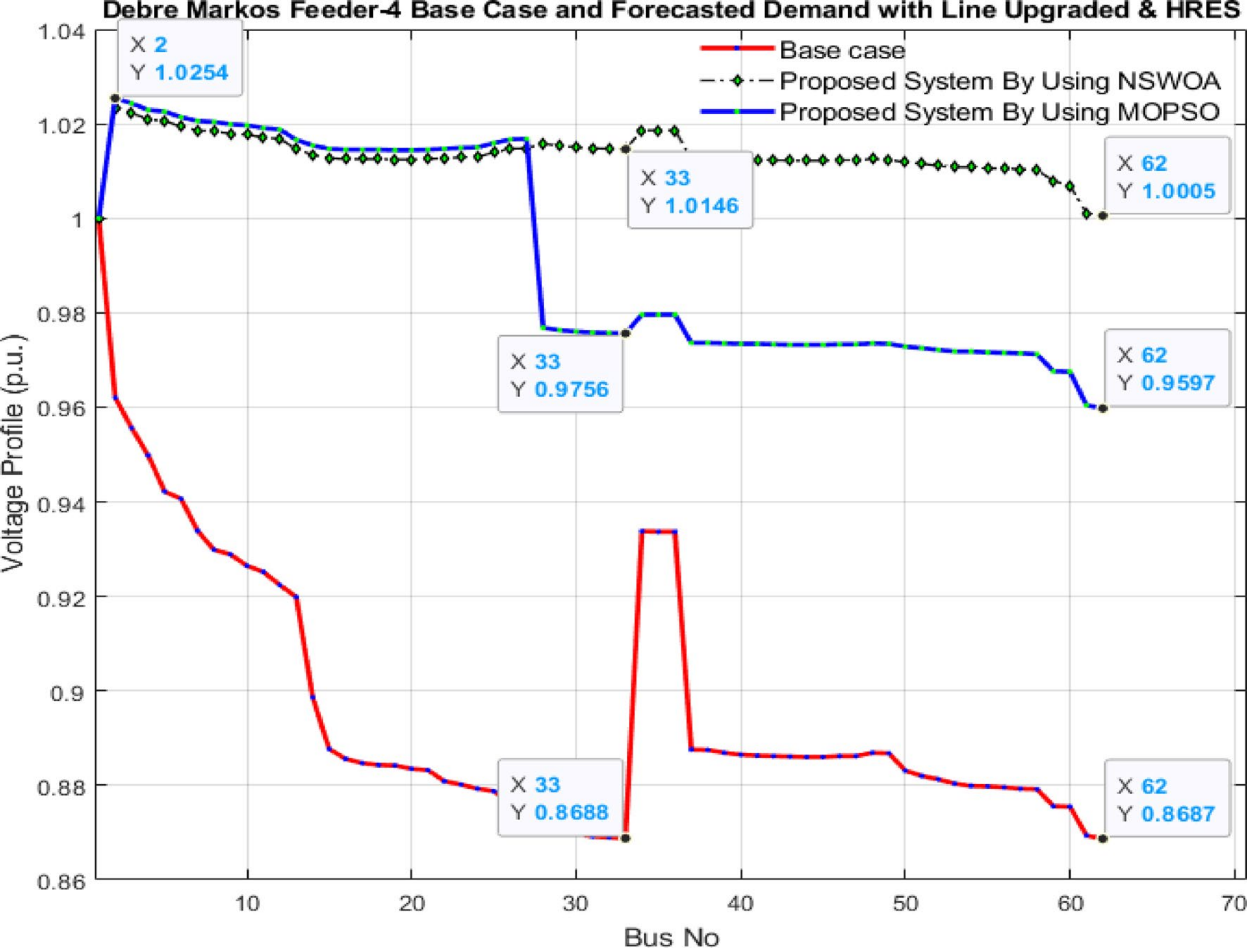
Comparison of voltage profile for the three cases (Base case, HRES by MOPSO, and HRES by NSWOA).

As presented in Table 5, the reduction in the total voltage deviation and power loss by using NSWOA was 96.062% and 94.964%, respectively. Whereas by using MOPSO, the total power loss and voltage deviation were 94.743% and 91.942%, respectively. According to this result, the reduction achieved by using NSWOA for total power loss and voltage deviation was 25.091% and 37.502% better than that achieved using MOPSO. The node voltage profile is also compared, as shown in Fig. 9, using NSWOA and MOPSO techniques.

Based on Fig. 9, it is evident that the voltage profile of the system, when employing NSWOA, is superior to that obtained through MOPSO. This figure shows that the results obtained utilizing NSWOA are close to but not quite equal to unity. Where the voltage profile gets to unity, and the voltage deviation becomes zero.

### Proposed system cost effectiveness comparison with existing system

The technological and economic viability of using hybrid systems to supply power to the selected distribution network has been examined in this section. The economic efficiency and greenhouse gas emissions of the proposed hybrid system were compared to those of the existing system (Grid + diesel generator (DG)) and only the DG power generation system. Following the simulation of the options utilizing NSWOA optimization techniques, the best configurations were found. The cost and GHG emission effectiveness of the proposed hybrid renewable power generation with respect to the existing system (Grid + DG) and only DG scenarios were presented in Table 6.

**Table 6:** Results of cost and GHG emission on the proposed hybrid, existing (Grid + diesel generator) and only diesel generator scenarios.

Parameters		Scenarios		
		Proposed HRES system	Existing (Grid + DG) system	Only DG system
Financial	COE (€/kWh)	0.08126	0.08752	0.12008
	LCC (€)	4.5071 × 10^6^	4.9572 × 10^6^	7.2146 × 10^6^
GHG	CE (kgCO_2_)	8.7536 × 10^6^	1.6122 × 10^7^	10.6875 × 10^7^

As presented in Table 6, the COE (€/kWh), LCC (€), and GHG emissions (kg CO_2_) parameters were recorded in deferent scenarios, such as the proposed hybrid system, existing system (Grid + DG), and only DG power generation system.

In comparison, the best combinations are solar PV/biogas/PHES/SMES with a COE of 0.08126 €/kWh, an LCC of 4.5071 × 10^6^ €, and emission of 8.7536 × 10^6^ kgCO_2_/year. The national grid with DG system and only DG had a COE of 0.08752 and 0.12008 €/kWh, a LCC of 4.9572 × 10^6^ and 7.2146 × 10^6^ €, and 1.6122 × 10^7^ and 10.6875 × 10^7^ kgCO2 emissions annually, respectively. It was discovered that the COE, LCC, and GHG emissions of these suggested configurations were lower than those of the existing (Grid + DG) and only DG systems.

This result indicates that when the proposed hybrid renewable power generating system scenarios are implemented, the optimum outcome for COE is less than 7.153% in the existing system and 27.115% in the only DG system. Meanwhile, the LCC achieved 9.081% and 37.528%, respectively, less than that of the current system (Grid + DG) and only DG scenarios. Furthermore, comparing the proposed system to the existing setup (Grid + DG) and the only DG scenario, the decrease in greenhouse gas emissions was 45.704% and 91.809% higher, respectively.

**Figure 10:**
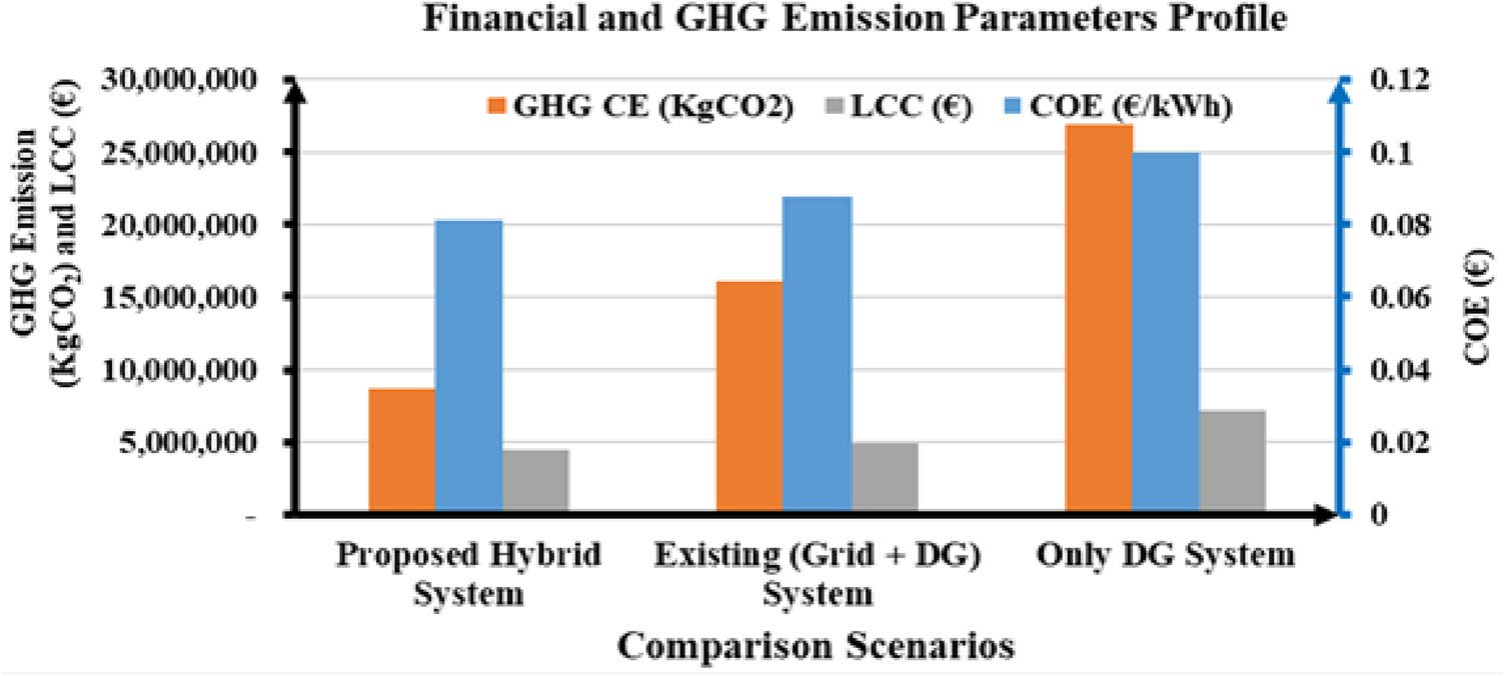
Graphical representation of cost and GHG emission on the proposed hybrid, existing (Grid + diesel generator) and only diesel generator scenarios.

The proposed system constraints’ numerical values and the participation rate of renewable energy generation with energy storage systems significantly influence financial and environmental effectiveness. Figure 10 illustrates the financial parameters (COE and LCC) and GHG emission effectiveness for the proposed hybrid system, existing (grid + diesel generator), and only diesel generator scenarios.

In accordance with the three scenarios that are mentioned in Table 6, which include the proposed HRES system, the existing system (grid + diesel generator), and the only diesel generator system, Fig. 10 demonstrates that the proposed HRES system scenario is optimal for the values of financial parameters such as COE and LCC, as well as the effectiveness of greenhouse gas emissions. Further, the diesel generator scenario is the only one that has the worst numerical values of financial metrics, including COE ((€/kWh) and LCC (€), as well as GHG emission (KgCO_2_).

## Conclusion and future research directions

Electricity plays a pivotal role in driving a nation’s economic prosperity, fostering advancements in technology, and enhancing the quality of life for its citizens. However, persistent power shortages pose significant challenges, leading to disruptions in daily life and hindering economic development. In Ethiopia’s Debre Markos distribution network, frequent power outages, averaging over 800 h annually in the past 5 years, have necessitated the deployment of diesel generators to mitigate the impact on businesses and households. Nonetheless, reliance on these generators comes at a considerable cost, both economically and environmentally, exacerbating the strain on limited resources. Addressing these challenges requires strategic improvements in infrastructure and energy management practices to ensure reliable power supply, minimize pollution, and support sustainable development initiatives.

In conclusion, this paper proposes a solution to the challenges faced by the Debre Markos University’s distribution system through the introduction of a grid-connected hybrid solar-biogas power generation system, supplemented by an SMES–PHES energy storage system. The study undertook a thorough examination of the existing and projected demand within the distribution network using a backward/forward sweep load flow method. The analysis revealed that the voltage profile of the network fell below the minimum standards set by the IEEE, while total power loss peaked. Consequently, measures were implemented to improve the system’s performance and address these issues effectively. The suggested approach was implemented by utilizing various metaheuristic optimization techniques, taking into consideration several objective functions and constraints. When applied to three objective functions (i.e., financial, reliability, and GHG emission), the results from the whale optimization algorithm were better than other metaheuristic optimization techniques. Based on the findings shown in the results section, it can be concluded that triple objective functions exhibit superior performance compared to alternative approaches. This is attributed to their comprehensive assessment of financial, technical viability (reliability), and environmental parameters. Furthermore, by applying forward/backward sweep load flow analysis, the integration of the suggested system led to appreciable enhancements in the distribution network’s impact. This was evident in the reduction of total power losses from 470.78 to 18.54 kW and voltage deviation from 6.95 to 0.35 p.u., as well as the fact that the voltage profile of the distribution system is swing-between 1 and 1.0234 p.u., which now comply with the standards set by the IEEE. A study was also conducted to determine how much the proposed hybrid system would cost and how well it would reduce greenhouse gas emissions compared to other (only DG) scenarios and existing systems that use both grid power and DG as a backup. Based on this finding, the optimum COE in the existing system is less than 7.153%, and in the only DG system, it is less than 27.115% when the proposed hybrid system scenarios are applied. However, the LCC’s results were lower than those of the existing system (Grid + DG) and only DG scenarios, at 9.081% and 37.528%, respectively. Moreover, the reduction in greenhouse gas emissions was 45.704% and 91.809% higher, respectively, when comparing the proposed system to the current configuration (Grid + DG) and the sole DG scenario. The results revealed that the proposed system is the most cost-effective and has the lowest GHG emissions among the alternatives considered.

While considering future research initiatives, there are various interesting paths that may be taken to further enhance the integration of HRES into distribution networks. Utilizing modern optimization methods, such as artificial intelligence (AI) and machine learning (ML) approaches, can improve the efficiency and dependability of integrating HRES by optimizing the design and operation of the system in dynamic and uncertain environments. Furthermore, the examination of demand-side management tactics and smart grid technologies can enhance the efficiency of energy consumption patterns, diminish peak demand, and ultimately optimize the exploitation of renewable energy resources. Furthermore, it is essential to evaluate the ability of HRES-integrated distribution networks to withstand and adapt to different disruptive events, such as severe weather and cyber-attacks. This assessment should include the development of strategies to enhance resilience, such as configuring microgrids and implementing adaptive control mechanisms. These measures are necessary to guarantee a consistent and dependable power supply. Furthermore, performing thorough techno-economic assessments and including stakeholders in policy frameworks can offer valuable insights into the long-term cost-efficiency and sustainability of deploying HRES. This, in turn, can make it easier to adopt and integrate HRES into the current electricity infrastructure. Furthermore, it is crucial to perform environmental impact assessments in order to analyze the ecological impact of deploying HRES and to identify strategies to mitigate any negative environmental consequences. This is vital for supporting the growth of sustainable energy and reducing harmful impacts on the environment. By following these research paths, stakeholders can collaboratively promote the incorporation of renewable energy systems into distribution networks, so contributing to worldwide endeavours for a low-carbon future.

## Data Availability

The datasets used and/or analysed during the current study available from the corresponding author on reasonable request.
